# Survival pattern of rare histological types of breast cancer in a Nigerian institution

**DOI:** 10.11604/pamj.2019.34.114.16925

**Published:** 2019-10-29

**Authors:** Atara Isaiah Ntekim, Ayorinde Mobolanle Folasire, Musa Ali-Gombe

**Affiliations:** 1 Department of Radiation Oncology, College of Medicine, University of Ibadan, Ibadan, Nigeria; 2 Department of Radiology, Gombe State University, Gombe, Nigeria

**Keywords:** Breast, cancer, rare, histology, survival, Nigeria

## Abstract

**Introduction:**

breast cancer is the most common cancer affecting women worldwide. It is a heterogeneous disease with diverse histological types that are associated with different natural history and response to therapy. Invasive ductal and lobular carcinoma are the most common histological types. There are rare histological types with different biological behaviours from the common types, although treatment approaches are the same. Data on rare histological types of breast cancer in our population are scarce raising the need to identify these patients and document their treatment outcome. The objectives of this study are to determine the proportion and treatment outcomes of breast cancer patients with rare histological types.

**Methods:**

this was an observational retrospective study using records of patients treated for breast cancer at the University College Hospital Ibadan Nigeria from 2008 to 2012. Patients with rare histological types were selected for further analysis. Data on patient and tumour characteristics were extracted and five-year survival pattern was determined using Kaplan Meier method.

**Results:**

the total number of patients with breast cancer was 761. Thirty-two (4.2%) had rare histology that consisted of medullary carcinoma 14(1.9%), mucinous carcinoma 10(1.4%) and 2(0.3%) each for squamous cell carcinoma, stromal sarcoma, cribriform carcinoma and Paget's disease. The overall five-year survival was 50% with median survival of 52 months.

**Conclusion:**

the proportion of breast cancer patients with rare histology is low similar to other reports among Caucasians. Medullary adenocarcinoma was the most common subtype followed by mucinous adenocarcinoma.

## Introduction

Breast cancer is a heterogeneous disease that consists of several distinct entities with different features. One of these is its various histological types. The World Health Organization (WHO) classifies breast cancers into epithelial (e.g. invasive ductal carcinoma, invasive lobular carcinoma, metaplastic carcinoma, medullary carcinoma, etc.), mesenchymal tumours (such as angiosarcoma, osteosarcoma, liposarcoma, leiomyosarcoma etc.), fibro-epithelial tumours (phylloides tumour, periductal stromal sarcoma), malignant lymphoma (diffuse large B-cell lymphoma, Burkitt’s lymphoma etc.) and metastatic tumours [1]. Other classes consist of tumours of the nipples, male breast cancer and myo-epithelial tumours. About 75% of breast cancers are invasive ductal carcinoma while invasive lobular carcinoma comprises about 5-15% and the other (rare) types comprise less than 10% of breast cancers [2]. Rare histological types of breast cancer are therefore all other histological types that constitute only about 10 percent of all breast cancers while the predominant types are invasive ductal and invasive lobular carcinomas (constituting about 90 percent).The various histological types are associated with varying epidemiology, diagnostic issues, clinical presentation and prognosis. Invasive ductal carcinoma and invasive lobular carcinoma, being the predominant histological types, have been widely studied and there are guidelines towards their management. Depending on the hormonal and immunohistochemical status, adjuvant chemotherapy, hormonal therapy and anti HER-2 positive agents are recommended as appropriate. On the other hand, there are no treatment guidelines for patients with rare histological types of breast cancer. This is probably because their rarity makes it difficult to organise clinical trials with sufficient sample sizes and timely accrual of subjects. At present, these tumours are being managed with standard therapy as there are no evidence based specific methods for their management [3]. The relatively small number of patients with breast tumours other than the ductal, lobular or mixed ducto-lobular types, has reached numbers that cannot be ignored due to the increase in the incidence of breast cancer and the associated uncertainty in its outcome. We believe that there is paucity of data about these rare malignant breast tumours in our population and this study will assist in the understanding of these unique subgroups and identifying special requirements for their management. The aim of the study was to determine the treatment outcome of breast cancer patients with rare histology types in the study population. To achieve this aim, this study was carried out with the objectives of determining the proportion of breast cancer patients with rare histological types, the clinical and treatment characteristics of patients with rare histological types of breast cancer and the five-year survival pattern of the patients with rare histological types of breast cancer in the study population.

## Methods

The study was carried out at the Radiation Oncology Department, University College Hospital (UCH), Ibadan Nigeria. This was a retrospective cross-sectional study of all patients with rare histological types of breast cancer treated at the Radiation Oncology Department, UCH from January 2008 to December 2012. The year 2012 was the cut off year so that the last patient could have follow up data of up to five years. Patients included were females with histologically confirmed breast cancer who received optimal treatment. Patients were considered to have optimal treatment if they had surgery, chemotherapy and radiotherapy to the breast/chest wall. Patients without histologic diagnosis, and those with histologic diagnosis of invasive ductal carcinoma or invasive lobular carcinoma were excluded. Ethical approval was obtained for this study from the institution's Ethical Review Committee.

**Data collection**: all available hospital records of breast cancer patients seen from 2008 to 2012 were retrieved. Patients with rare histological types of breast cancer were selected for further analysis. Information obtained from hospital records and radiotherapy treatment records consisted of patients' biodata including age at presentation and patients' and relatives' telephone numbers. Duration of illness, site of tumour, and menopausal status at diagnosis were also obtained. Pathological features of the disease such as histological type, stage at presentation and nodal status, were also documented. These were determined from the history, physical examination and investigations during pre-treatment evaluation. The details of treatment received were also noted. These included radiotherapy, type of surgery (breast conserving or mastectomy), axillary clearance, chemotherapy regimen, and hormonal therapy. The status of patients was determined at two and five years after diagnosis. The time of commencement of treatment, time of first loco regional recurrence and/or metastasis after completion of treatment were also noted. The status was either survival beyond end of study, loss to follow up, or death. Patients who were lost to follow up, were contacted using their telephone numbers or that of their relatives which was recorded in the case notes in order to ascertain their current status and if they were dead the date of death was also obtained.

**Data analysis**: socio-demographic, clinical and treatment variables of patients were presented in tables, pie charts and bar charts using frequency, percentages, mean, range and standard deviation. Survival curves were calculated using Kaplan Meier method. The patient status as of December 2017 was categorized as alive, dead and lost to follow-up. The median survival times were obtained from the Kaplan Meier survival curve. The disease-free survival at 2 and 5 years were also determined, so also time to recurrence. The data were analysed using SPSS version 21.0 Software.

## Results

**Figure 1:**
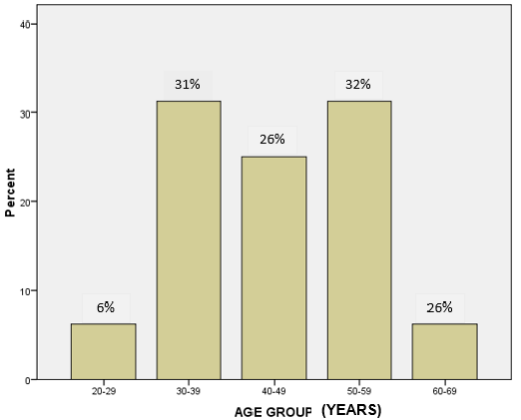
Age distribution of breast cancer patients with rare histological types

**Socio-demographic characteristics**: a total of 761 patients with breast cancer were seen during the study period. Out of these 32(4.2%) patients had rare breast cancers and were analysed. All were females with ages ranging from 28 to 62 years with a mean age of 44.6 ± 10.3 years. There were bimodal age range peaks at age ranges 30-39 and 50-59 years. The age group distribution of the patients is shown in Figure 1. Twenty-two (68.8%) out of the 32 patients studied were premenopausal.

**Figure 2:**
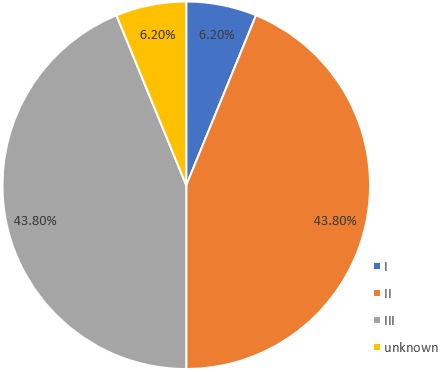
Distribution by stage at presentation of breast cancer patients with rare histological types

**Clinical characteristics and treatment received by patients**: histological types consisted of medullary carcinoma 14(1.9%), mucinous carcinoma 10(1.4%) and 2(0.3%) each for squamous cell carcinoma, stromal sarcoma, cribriform carcinoma and Paget's disease out of the total breast cancer cases seen. The mean duration of illness was 26.6 ± 36.9 months with a range of 1 to 144 months. Fourteen patients presented in stages II and III respectively making a total of 87.6% (43.8% each). No patient presented at stage IV although the stage at presentation for 2(6.2%) patients were not known. Stage distribution is shown in Figure 2 while clinical characteristics and treatment received are presented in Table 1.

**Table 1:** Clinical characteristic and treatment received by breast cancer patients with rare histological types (N=32)

Variable	Frequency	Percentage (%)
**Tumour site**		
Right	18	56.25
Left	14	43.75
**Clinical axillary lymph node**		
Non palpable	14	43.75
Palpable	18	56.25
**Type of surgery**		
Breast conserving	6	18.75
Mastectomy	26	81.25
**Axillary clearance**		
No	16	50
Yes	16	50
**Chemotherapy regimen**		
CAF	10	31.25
CMF	10	31.25
AC	10	31.25
Taxanes	2	6.25

CAF: cyclophosphamide, Doxorubicin, 5-Fluorouracil; CMF: cyclophosphamide, Methotrexate, 5-Fluorouracil; AC: Doxorubicin, Cyclophosphamide

**Figure 3:**
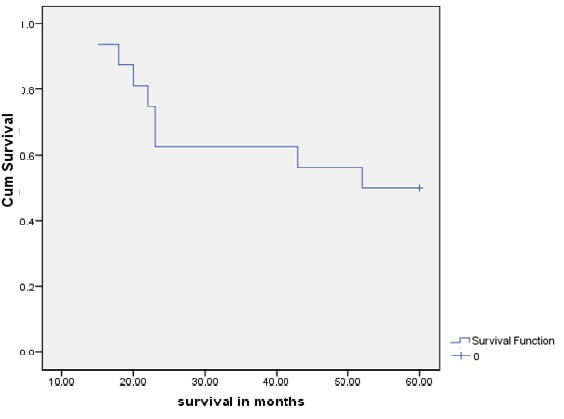
Overall survival of breast cancer patients with rare histology (Kaplan-Meier curve)

**Survival**: the overall two- and five-year survival rates in this study were 62.5% and 50% respectively. The overall two- and five-year Kaplan-Meier curves are shown in Figure 3. Two patients (6.2%) were lost to follow up. The median survival time at five years was 52.0 months (Figure 3).

**Figure 4:**
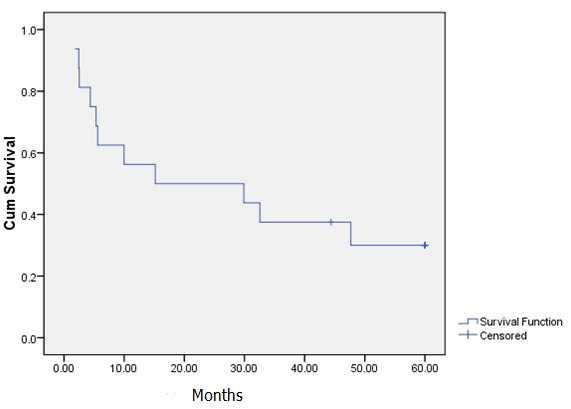
Disease-free survival of breast cancer patients with rare histology (Kaplan-Meier curve)

**Recurrence**: the 2- and 5-year disease-free survival were 50.0% and 31.2% respectively. The mean time to recurrence was 16.8 months ± 1.7 months with a range of 1.8-47.6 months. The 2- and 5-year disease-free survival Kaplan-Meier curves are shown in Figure 4. The demographic and clinical characteristics with survival pattern based on histological types of the patients are presented in Table 2. The table shows that patients with medullary histology were the highest followed by mucinous histology.

**Table 2:** Demographic and clinical characteristics with survival pattern of patients with rare breast cancer based on histology (N=32)

	Medullary	Mucinous	Others
No. of patients Mean age(years)	14 (43.8%) 43.7 (±12.1)	10 (31.2%) 47.4 (±10.3)	8 (25.0%) 42.8 (±8.5)
**Menopausal status**			
*Premenopausal*	10 (71.4%)	6(60.0%)	6(75.0 %)
*Postmenopausal*	4(28.6%)	4 (40.0%)	2 (25.0%)
Stage I	2 (14.3%)	0	0
II	8 (57.1%)	4 (40.0%)	2 (25.0%)
III	4 (28.6%)	6 (60.0%)	4(50.0%)
IV	0	0	0
Unknown	0	0	2(25.0%)
Palpable axillary node	6 (42.9%)	6 (60.0%)	6 (75.0%)
**Status at 5 years**			
*Alive*	8 (57.1%)	6 (60.0%)	2 (25.0%)
*Dead*	4 (28.6%)	4 (40.0%)	6 (75.0%)
*Lost to follow up*	2 (14.3%)	0	0
Median survival	60.0	not reached	5.0
(Months)	(95% CI 2.6-117.4)		(95% CI 0.0-17.7)

## Discussion

Rare breast carcinomas are reported to be less than 10% in most literature [2]. In this study they accounted for 4.2% which is in line with above report. In the present study, medullary carcinoma constituted 1.9% of the total breast cancers seen under the period of study, it has been reported to be about 1-7% in most cases depending on the strictness of diagnostic criteria used [1,4]. With respect to mucinous carcinoma, it accounted for 1.4% which is close to the 2% that has been reported in the literature [1], although in some reports mucinous carcinomas represented up to 4% of all breast cancers [5]. The most common type of breast cancer with rare histology from this study was medullary carcinoma, however, mucinous carcinoma has been reported to be the most common type of rare breast cancers in other populations [2]. According to the National Cancer Institute data, mucinous carcinoma is the main "rare" form of breast cancer comprising 49.2% of rare breast cancers and 4.3% of all breast cancers [2]. Cribriform carcinoma is said to account for 3.5% of breast cancers [6], while in some other studies it is as low as 0.1%-0.6% [7] which is consistent with this study (0.3%). We reported only two cases (0.3%) of primary squamous cell carcinoma of the breast. From literature the incidence of squamous carcinoma of the breast has been reported to be between 0.06 and 0.2% of all breast cancers [8]. Paget disease amounted to about 0.3% in our findings which is lower than the incidence of 1-4.3% that was found in other reports [1]. Stromal sarcoma was also 0.3% which is similar to the incidence of 0.3-1.0% reported in Western countries [1].

In our findings, the age range of patients with rare breast histological types was between 28.0-62.0 years with a mean age of 44.6 years. There were bimodal age range peaks (30-39 and 50-59) and 68.8% of the patients were premenopausal. The age range of women with medullary carcinoma as reported in another study was between 45-52 years [9], while the age range of 53-58 years was reported for cribriform carcinomas [2]. Gudaviciene and colleagues (2014), documented a mean age of 53.99 (± 14.74) for medullary carcinomas and 63.48 (±13.75) for mucinous carcinomas [6]. Another study also reported that mucinous carcinomas present at the oldest median ages (71 years) [10]. Phyllodes tumours are known to occur predominantly in middle-aged women (average age of presentation is 40-50 years) and in Asian countries they occur at a younger age (average 25-30 years) [1]. However, malignant phyllodes tumours develop on average 2-5 years later than benign ones [1]. The two stromal sarcomas in our study were aged 36 and 45 years respectively. These variations in the age range of patients from this report are likely due to population differences in the disease process as all other data on age at diagnosis were from Caucasian and Asian populations. In contrast to the large number of premenopausal women in our study, Gudaviciene and colleagues (2014) reported that only six (42.9%) out of the fourteen patients with medullary carcinoma were premenopausal and fifteen (24.6%) out of sixty-one patients with mucinous carcinoma were premenopausal. The large number of premenopausal women in our study may be attributed to the observations that higher proportion of triple negative breast cancer generally occur in younger women in Africans compared with Caucasians [11].

Like in most Nigerian studies, patients' presentation to the healthcare services was delayed. The mean duration of symptoms for this cohort was 26 months which is longer than the 12.2 months reported in a Nigerian study for all breast cancers [12]. Reasons for late presentation have been suggested to be fear of mastectomy, ignorance, spiritual belief and preference for alternative therapies [12]. Another reason can be that most of these rare breast cancers are slow growing which may not disturb the patient much to prompt coming to hospital early [13]. In terms of overall staging in this study, a significant number of the patients (43.8%) presented in stage II and an equal proportion (43.8%) also presented with stage III (Table 2). When compared with reports from local studies which assessed all breast cancer subtypes, this group of patients (with rare histology) have a higher number of patients presenting at early stages (43.8% versus 6.2%) [14,15]. Worthy of note is that none of the patients presented at stage IV. Patients with medullary carcinoma presented in early stages in this study with 14.3% in stage I and 57.1% in stage II with the remaining 28.6% presenting with stage III disease. Although these patients presented with relatively earlier stages of disease than in most local reports on all breast cancer histology types, the proportions are still lower than what was found in a Western study where patients with medullary carcinoma had 35.7% presenting in stage I, 64.3% in stage II and none presented in stage III or IV [6]. In this present study, forty percent of patients with mucinous carcinomas presented in stage II while the remaining 60.0% presented with stage III disease. In the above-mentioned study by Gudaviciene and colleagues (2014), 27.4% of medullary carcinomas presented at stage I, 53.2% at stage II, 16.1% at stage III and 3.2% at stage IV. The studies from the Western populations had more patients presenting early than the present study. Palpable axillary nodes were documented in 56.2% of our patients which is lower than 87.6% reported among breast cancer patients with all histological types in Nigeria [16]. In our cohort 42.9% of medullary carcinomas had palpable axillary lymph nodes and none had palpable supraclavicular nodes. With respect to mucinous carcinomas, 60.0% had palpable axillary lymph nodes while none had palpable supraclavicular node. Among the other subtypes 75.0% had axillary nodes. These are quite high when compared with Gudaviciene *et al'*s study, where mucinous and medullary carcinoma represented 25.8% and 28.6% of the study population, respectively [6] Another study reported 27.0% for medullary, 3.0-15.0% for mucinous and 14.0% for cribriform carcinomas [2].

The management of these rare subtypes in our study did not differ from the treatment given to the major types of breast cancer. Rare histological types often display peculiar clinical behaviours, especially as they are hormone receptor negative. There are no randomised clinical trials on the extensive clinical evaluation of these groups due to the small number of patients affected. Most of the information on outcome and treatments are derived from small series and case reports, and therefore there are no clear guidelines on treatment recommendation. Guidelines commonly used are the same used for the major subtypes of breast cancer. The only exception is secretory carcinoma which can arise in childhood and breast-conserving surgery represents a dilemma since it is better to avoid radiotherapy in such cases [17]. However, the National Comprehensive Cancer Network (NCCN) USA Guidelines now has specific treatment recommendations for favourable tubular and mucinous subtypes on neoadjuvant chemotherapy [18]. The overall two- and five-year survival rates in this study were 62.5% and 50% respectively. This study reported better outcomes compared to other studies from Nigeria on all breast cancer subtypes. For example Popoola and colleagues (2012) reported a 5 year overall survival of 25.6% which is about half of what is reported in this study [16]. Similarly, Kene and colleagues also reported that only 24.5% of patients with advanced breast cancer survived beyond 30 months [19]. The median five-year survival time in this study was 52.0 months. This is also high when compared with 31 months reported for all breast cancer histology subtypes in Nigerian studies [16,20]. For individual histology, we also found that the 5-year survival rate was 57.1% for medullary and 60.0% for mucinous carcinomas while other types had a combined 25.0% 5-year survival rate. The median survival time was 60.0 months for medullary and 5.0 months for other types. At 5 years the median survival time for mucinous carcinoma was not reached (Table 2). The survival of most of the rare histological types reported around the world is high compared with values for patients with invasive ductal or lobular carcinomas. The 5-year breast cancer-specific survival rate for mucinous carcinoma has been reported as 94% compared with 82% for infiltrating ductal carcinoma [21]. Similarly, medullary carcinoma has also been reported to have a better prognosis than invasive ductal carcinoma, even though this has been disputed by some authors [2]. The overall 10-year survival for medullary carcinoma varies from about 50% to more than 90%. Such disparity can be attributed to differences in diagnostic criteria [22]. In another study, 10-year overall survival was 85% for medullary carcinoma as compared to 68% in patients with invasive ductal carcinoma [23]. The mean survival time in years for medullary and mucinous carcinomas has been reported as 8.26 years (SD±2.91) and 6.61 years (SD±2.29) respectively. All the above support the findings of relatively better survival in this study compared to studies involving invasive ductal/lobular carcinomas in our environment.

The ten-year overall survival of cribriform carcinoma has been reported to be about 90-100% [2]. Primary squamous cell carcinoma of the breast carries a poor prognosis. Hennessey and colleagues (2005) reported that patients with localized squamous cell carcinoma at diagnosis were found to have poor overall 5 year survival of 64.0% in the Surveillance, Epidemiology, and End Results (SEER) database and even a lower value 40.0% in a single- institution series [24]. It has been found that Paget's disease of the breast, even if it involves only the nipple at presentation, is associated with an underlying breast carcinoma in 98.5% of the cases [25]. The prognosis of Paget's disease is dependent on the underlying invasive ductal or lobular malignancy. The 10-year overall survival rate in a study for Paget's disease of the breast with invasive carcinoma was 49%, whereas those with invasive carcinoma without Paget disease had 64% survival rate [25]. These figures further explain the low survival seen in the "other" group in this study which consists of squamous cell carcinoma, Paget's disease, cribriform carcinoma and stromal sarcoma. The 2 and 5-year disease free survival rates in this study were 50.0% and 31.2% respectively, with a mean time to recurrence of 16.8 months (SD±1.7). This indicates a high recurrence rate which is one of the distinctive findings from this study. This rate is low when compared with a case series of 46 cases of medullary carcinoma which reported a 10-year-distant relapse-free survival of 95% [22]. Lymph node involvement, which is low in mucinous carcinoma is associated with a significantly worse 5-year disease free or overall survival [26]. Patients in this study had a high number of lymph node involvement (Table 2) which can possibly explain the high rate of disease recurrence.

## Conclusion

Rare breast cancer subtypes are a heterogeneous group of malignancies with different clinical behaviours and outcomes. In our environment they present at a younger age group mostly among premenopausal women. Medullary histology was the predominant subtype in this series. Higher survival rates were noted when compared with the major histological types described in our environment even though the survival is still low when compared with values from high income countries. In line with other local studies for all breast cancer subtypes the disease free interval is still low.

### What is known about this topic

- Breast cancer with rare histology forms about 10% of breast cancers among Caucasians;
- Breast cancer is associated with high mortality in Nigeria;
- Lymph node involvement confers poor prognosis in all histological types of breast cancer.

### What this study adds

- Prevalence of breast cancer with rare histological types was 4.2%;
- Medullary carcinoma was the most common type followed by mucinous type;
- The two and five year survival rates were 62.5% and 50% respectively.

## Competing interests

The authors declare no competing interests.
